# Supplementary material for: Resolving the full spectrum of human genome variation using Linked-Reads
Source: Genome Res. 2019 Apr;29(4):635–45. doi: 10.1101/gr.234443.118 (PMC6442396; doi:10.1101/gr.234443.118)
Supplement: Supplemental Material [file supp_gr.234443.118_Supplemental_longranger-2.2.zip › longranger-2.2.tar.bz2!/lr2.2-public/lib/stan/cmdstan/stan/doc/api/html/functions_vars_v.html]

Stan: Class Members - Variables


|  |  |
| --- | --- |
|  | Stan  2.5.0  probability, sampling & optimization |


- Main Page
- Related Pages
- Namespaces
- Classes
- Files

- Class List
- Class Index
- Class Hierarchy
- Class Members

- All
- Functions
- Variables
- Typedefs
- Enumerator
- Related Functions

- \_
- a
- b
- c
- d
- e
- f
- g
- h
- i
- k
- l
- m
- n
- o
- p
- q
- r
- s
- t
- u
- v
- w
- x
- y
- z


All Classes Namespaces Files Functions Variables Typedefs Enumerations Enumerator Friends Macros Pages

### - v -

- V
  : stan::mcmc::ps\_point
- val\_
  : stan::agrad::fvar< T >
  , stan::agrad::vari
  , stan::gm::double\_literal
  , stan::gm::int\_literal
- var\_adaptation\_
  : stan::mcmc::stepsize\_var\_adapter
- var\_decl\_r
  : stan::gm::var\_decls\_grammar< Iterator >
- var\_decls\_g
  : stan::gm::program\_grammar< Iterator >
  , stan::gm::statement\_grammar< Iterator >
- var\_decls\_r
  : stan::gm::var\_decls\_grammar< Iterator >
- var\_dims\_
  : stan::gm::assignment
- var\_lhs\_r
  : stan::gm::statement\_grammar< Iterator >
- var\_map\_
  : data\_only\_expression
  , stan::gm::bare\_type\_grammar< Iterator >
  , stan::gm::contains\_nonparam\_var
  , stan::gm::contains\_var
  , stan::gm::expression07\_grammar< Iterator >
  , stan::gm::expression\_grammar< Iterator >
  , stan::gm::functions\_grammar< Iterator >
  , stan::gm::program\_grammar< Iterator >
  , stan::gm::statement\_2\_grammar< Iterator >
  , stan::gm::statement\_grammar< Iterator >
  , stan::gm::term\_grammar< Iterator >
  , stan::gm::var\_decls\_grammar< Iterator >
- var\_resizer\_
  : stan::gm::dump\_member\_var\_visgen
- var\_size\_validator\_
  : stan::gm::dump\_member\_var\_visgen
  , stan::gm::generate\_init\_visgen
- var\_type\_
  : stan::gm::assignment
- variable\_
  : stan::gm::for\_statement
- variable\_r
  : stan::gm::term\_grammar< Iterator >
- varis\_
  : stan::agrad::precomputed\_gradients\_vari
- vector\_decl\_r
  : stan::gm::var\_decls\_grammar< Iterator >
- vi\_
  : stan::agrad::var
- vis\_
  : stan::agrad::op\_matrix\_vari
  , stan::agrad::op\_vector\_vari
- void\_return\_statement\_r
  : stan::gm::statement\_grammar< Iterator >


---

[ Stan Home Page ]

*© 2011–2014,
Stan Development Team.*
